# Answer to Dr. Jamison’s Case for Counsel

**Published:** 1888-03

**Authors:** F. H. Lutze

**Affiliations:** Cheshire, N. Y.


### ANSWER TO DR. JAMISON'S CASE FOR COUNSEL.

The homœopathic law is certainly never at fault; the lack of success is due either to the incurability of the case or to faulty selection of the remedy or to bad hygienic conditions, food, etc. And in a case where so much and many kinds of remedies have been taken it is often difficult to select the proper remedy at once, as each *not homœopathic remedy* given complicates the case.

There are in this case three great characteristic symptoms pointing to *Apis mel.* as the proper homœopathic remedy, viz.:

1. Stinging and burning pains.
2. Drowsy, sleepy, and worse after a good sleep.
3. The swelling.

I am positive that *Apis* will remove a great many of her symptoms if not entirely cure. I should give it a thorough trial, beginning with the sixth, perhaps, varying the potency or going gradually higher or lower, according to the result, a *dose morning and evening*, and give Sac. lac. as an intercurrent now and then.

Looking more closely into the case, I see that Apis covers a host of the lady's symptoms, present and past, as follows:

NOSE.—Catarrh (thick, white, or fœtid or bloody mucus, deep ulcers on tonsils and palate, causing, perhaps, ragged appearance of larynx by extension of ulceration). Nose red and swollen.

HEADACHE.—*Violent pain* in forehead and temples, left involving eye, with pain through orbit.

*Violent drawing pain* from back of neck extending behind left ear, and spreading over left half of head.

*Eyes burn like fire*, lachrymation scalding hot, lids very *sensitive to touch*.

Convulsions, trembling and jerking of limbs; œdema of the hands.

Walls of pharynx red.

Dyspnœa.

*Burning stinging pains* in the chest (left apex of lungs).

Vision dim (left eye). Violent pain in left knee; limbs swollen and numb; great inclination to sleep, *but cannot from nervous restlessness.*

Worse after a good sleep.

Menses scanty, dark, and clotted.

Dysmenorrhœa, fidgetiness (of feet).

The diagnosis is rather uncertain and difficult without more of the history of the attacks, but looks to me somewhat like "Petit Mal," complicated, perhaps, by allopathic drugging and time. Other remedies may be needed after Apis mel., as the symptoms will call for, perhaps Actea racemosa or Lachesis, Stannum, Stramonium, Theridion, but Apis should be given a thorough trial first, especially during the interval.

I have treated several young men for seminal emissions with Apis mel. and cured them.

In one of the most intractable ones of them all the symptoms were as follows:

Lascivious fancies, frequent emissions toward morning; the sight of a female or even animals in the streets aroused his passions and caused emissions; averse to company, especially of ladies; frequent headache; lazy, moody, constipated. Priapism, tall and robust, strong, robust, bony frame, etc., etc. Gave Nux v. and Staphisagria and Phos. in different potencies each, without any benefit apparently whatever.

Then complained of backache in lumbar region, better from motion and lying on something hard; gave Rhus tox$^{6}$ with some benefit, but soon got worse again on stopping the remedy.

Then said he must urinate very often, discharge smarting, especially at meatus. Urine profuse and pale, cloudy; almost constant discharge of a mass like the white of egg from penis; the same backache returned. Gave Apis mel.,$^{3}$ a dose three times a day, and reported *well* in three days; then gave Apis$^{30}$ for one week more and patient has remained well up to date, although it is nearly two years since treatment.

CHESHIRE, N. Y. F. H. LUTZE, M. D.

